# Transseptal Mitral Valve-in-Valve Implantation Using an Arteriovenous Loop

**DOI:** 10.1016/j.jaccas.2026.108146

**Published:** 2026-04-13

**Authors:** Yan-Jie Li, Lan Ma, Zi-Yong Hao, Xin Pan

**Affiliations:** a Department of Cardiology, Shanghai Chest Hospital, Shanghai Jiao Tong University School of Medicine, Shanghai, China; b Department of Ultrasonography, Shanghai Chest Hospital, Shanghai Jiao Tong University School of Medicine, Shanghai, China

**Keywords:** computed tomography, echocardiography, mitral valve, murmur, rheumatic heart disease, valve replacement

## Abstract

**Background:**

Transseptal mitral valve-in-valve (ViV) implantation is an established therapy for failed surgical bioprostheses. However, massive biatrial enlargement can preclude delivery, leading to procedural failure.

**Case Summary:**

A 75-year-old woman presented with heart failure after surgical mitral bioprosthesis implantation. Echocardiography revealed severe mitral bioprosthetic failure and massive biatrial enlargement. Computed tomography angiography showed a septal-annular angle of 106°. During the procedure, the stiff wire and the delivery system repeatedly prolapsed into the giant atrium. A full-body rail was created by snaring the guidewire from the femoral artery, providing adequate support. Then a 23-mm SAPIEN 3 valve was successfully deployed. Postprocedural mean gradient decreased to 4 mm Hg with mild paravalvular leak. The patient recovered uneventfully with significant functional improvement.

**Discussion:**

The full-body rail technique is a critical bailout strategy for mitral ViV in patients with massive atria, enabling coaxial alignment and safe delivery.

**Take-Home Messages:**

Massive biatrial enlargement and a horizontal septal-annular angle (>100°) can preclude standard delivery during mitral ViV implantation. The step-by-step creation of a full-body (arteriovenous) rail is a rescue technique to stabilize the delivery system.

## Visual Summary

**Figure:**
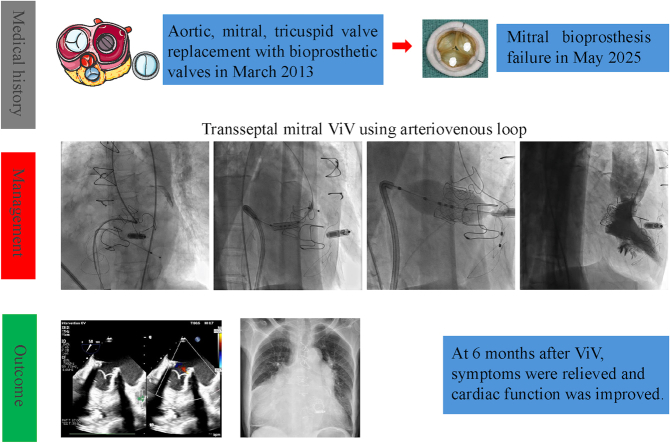

**Visual Summary:**
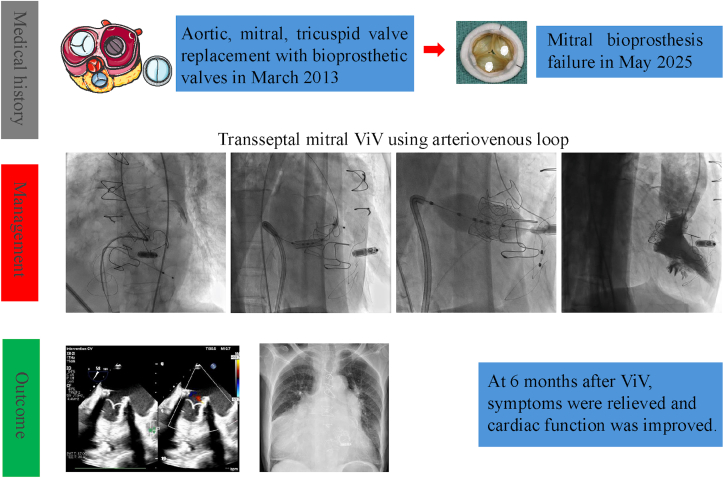
Graphic Representation of Valvular History, ViV Implantation, and Follow-Up ViV = valve-in-valve.

Mitral valve-in-valve (ViV) implantation is a safe and effective alternative to redo surgery in high-risk patients with degenerated bioprosthesis.^1^ The standard transseptal approach relies on adequate atrial support to direct the delivery system toward the mitral valve. However, in patients with massive biatrial enlargement (often seen in long-standing rheumatic heart disease or chronic atrial fibrillation), the atrial cavity provides no counterforce, causing the catheter or the valve system to prolapse. We report a case where this challenge was overcome by creating a full-body (arteriovenous) rail, enabling successful valve deployment.

**Take-Home Messages**

• Massive biatrial enlargement and a horizontal septal-annular angle (>100°) can preclude standard delivery during mitral valve-in-valve implantation.
• The step-by-step creation of a full-body (arteriovenous) rail is a rescue technique to stabilize the delivery system.

## History of Presentation

A 75-year-old woman (height 150 cm; weight 35 kg; body mass index 15.6 kg/m^2^) presented with a 2-week history of worsening dyspnea, orthopnea, and bilateral lower extremity edema. Physical examination during this admission revealed mild cyanosis of the lips, a 3/6 holosystolic murmur at the apex, and mild bipedal edema.

## Past Medical History

The patient had received a diagnosis of rheumatic heart disease (severe mitral stenosis, moderate-to-severe aortic stenosis, and severe tricuspid regurgitation with pulmonary hypertension) and atrial fibrillation in 2011. Then, she underwent surgery aortic, mitral, and tricuspid valve replacement and the Maze procedure in March 2013. During initial surgery, a standard transseptal approach was used. The interatrial septum was incised to expose the mitral valve. After completion of mitral valve replacement, the septal incision was closed primarily with a continuous suture. All replaced valves were bioprosthetic valves (Carpentier-Edwards Surgical Aortic Valve; Edwards Lifesciences): a 21-mm bioprosthesis in the aortic position, a 25-mm bioprosthesis in the mitral position, and a 29-mm bioprosthesis in the tricuspid position. In addition, she had a history of wireless pacemaker implantation in June 2023.

## Differential Diagnosis

When a patient presents with heart failure after bioprosthesis replacement, several etiologies must be considered. The differential diagnosis is primarily structural valve deterioration (SVD), non-SVD (pannus formation, paravalvular leak, and patient-prosthesis mismatch), prosthetic valve endocarditis, and thrombosis. On the basis of the timeline (12 years postimplantation), echocardiographic findings (leaflet tear and prolapse), and exclusion of infection or thrombus, the diagnosis was confirmed as SVD of the mitral bioprosthesis.

## Investigation

**Figure 1:**
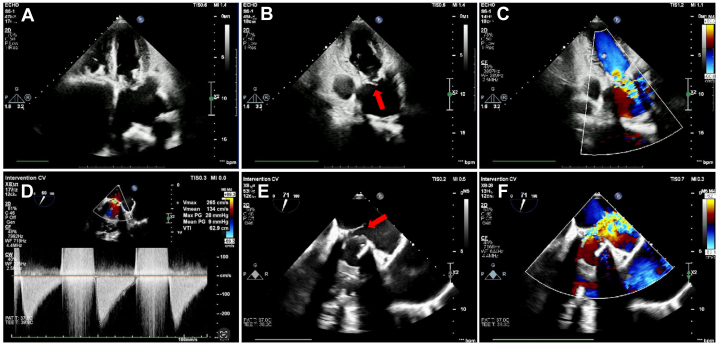
Transthoracic Echocardiography and Transesophageal Echocardiography Showing Degenerated Mitral Bioprosthesis (A) Massive biatrial enlargement. (B and C) Mitral bioprosthesis flail (arrow) with severe regurgitation on transthoracic echocardiography. (D) Mean transvalvular gradient of 9 mm Hg. (E and F) Bioprosthetic leaflet tear and prolapse (arrow) with severe intraprosthetic regurgitation on transesophageal echocardiography.

**Figure 2:**
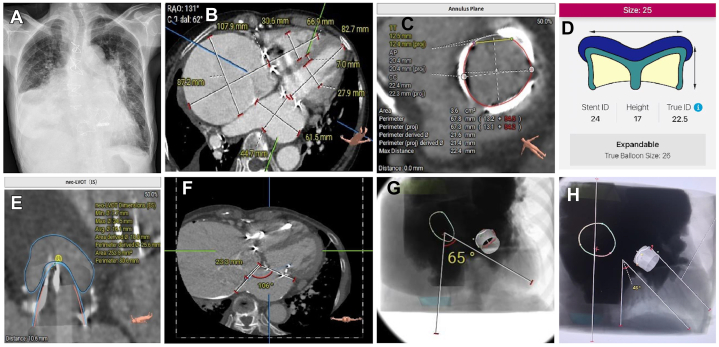
Procedural Challenge (A) Significantly increased cardiothoracic ratio. (B) Preprocedural computed tomography angiography showing the massive atrium. (C and D) True internal diameter of the bioprosthesis measured by computed tomography angiography and the baseline data. (E) Neo–left ventricular outflow tract area. (F) Septal-annular angle. (G) Angle between the inferior vena cava–atrial septum line and the mitral valve–left ventricular apex axis. (H) Angle between the inferior vena cava–superior vena cava axis and the mitral annulus–left ventricular apex line.

Laboratory investigations were notable for the following: pro–B-type natriuretic peptide, 8,562 ng/L; total bilirubin, 26.6 μmol/L; direct bilirubin, 6.4 μmol/L; alanine aminotransferase, 22 U/L; and aspartate aminotransferase, 35 U/L. The results of coronary computed tomography angiography were normal. Electrocardiography showed atrial fibrillation with ventricular demand pacing. Transthoracic echocardiography (TTE) showed massive biatrial enlargement (Figure 1A), with the right atrium measuring 107.9 × 87.2 mm and the left atrium measuring 51 × 61.5 mm. The mitral bioprosthesis showed severe regurgitation and a mean transvalvular gradient of 9 mm Hg (Figures 1B to 1D). The aortic and tricuspid bioprosthetic valves functioned well and pulmonary hypertension was present. Transesophageal echocardiography (TEE) confirmed bioprosthetic leaflet tear and prolapse, resulting in severe intraprosthetic regurgitation (Figures 1E and 1F, Video 1). Chest radiography showed a significantly increased cardiothoracic ratio (Figure 2A). Cardiac computed tomography angiography demonstrated the enlarged atrium (Figure 2B) and indicated that the true internal diameter of the bioprosthesis (bioprosthetic valve annulus area 360 mm^2^) corresponded to a 23-mm SAPIEN 3 valve together with the baseline data of prior bioprosthetic valve (Figures 2C and 2D). The simulated neo–left ventricular outflow tract area was 253.5 mm^2^ (Figure 2E), without risk of left ventricular outflow tract obstruction. Critically, the septal-annular angle was measured at 106° (Figure 2F), reflecting a near-horizontal orientation of the valve relative to the septum, exacerbated by the giant atria. At a transseptal puncture height of 2.5 cm, the angle between the inferior vena cava–atrial septum line and the mitral valve–left ventricular apex axis was 65° (Figure 2G). In addition, the angle between the inferior vena cava–superior vena cava axis and the mitral annulus–left ventricular apex line was 45° (Figure 2H).

## Management

The heart team deemed the patient at prohibitive risk for redo surgery (frailty, low weight, and previous cardiac surgeries). A transseptal mitral ViV procedure was planned.

**Figure 3:**
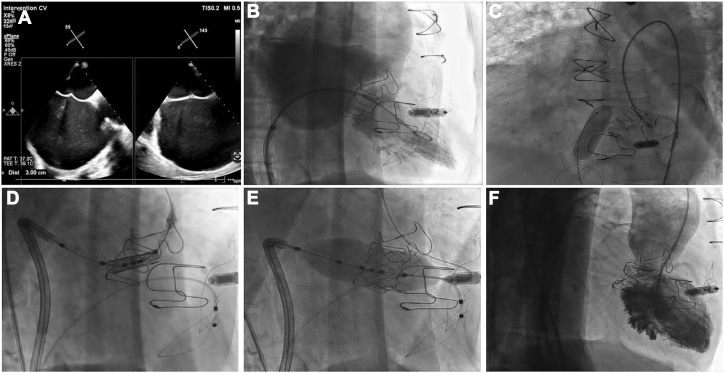
Mitral Valve-in-Valve Implantation (A) Final puncture height. (B) Left ventriculography showing massive mitral regurgitation. (C) Predilation using a 16-mm balloon with an arteriovenous loop. (D) Valve system advancing through the bioprosthetic valve. (E) SAPIEN 3 valve successfully deployed under rapid pacing. (F) Left ventriculography showing the well-deployed SAPIEN 3 valve with mild paravalvular leak.

Under general anesthesia, right femoral venous access was obtained. Transseptal puncture was performed under TEE guidance. Because of the massively enlarged right atrium, achieving a low puncture site was difficult; the final puncture height was approximately 3 cm above the annulus after repeated attempts (Figure 3A). Left ventriculography showed massive mitral regurgitation (Figure 3B, Video 2), with a left ventricular end-diastolic pressure of 13 mm Hg and a mean left atrial pressure of 27 mm Hg. Despite using a steerable catheter, the stiff wire could not be advanced into the left ventricle, let alone the delivery system.

**Figure 4:**
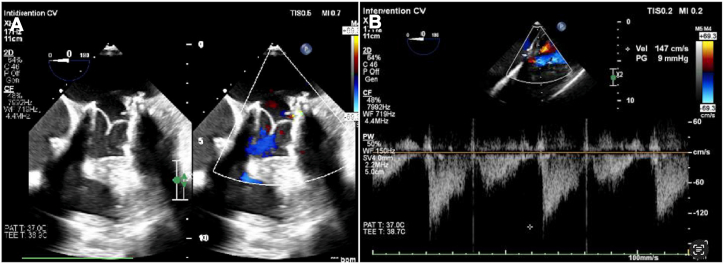
Final Result (A) Mild paravalvular leak on postdeployment transesophageal echocardiography. (B) Mean left ventricular outflow tract gradient of 9 mm Hg on postprocedural transthoracic echocardiography.

To overcome this anatomical hurdle, we converted the system into a “full-body rail”. A 260-mm hydrophilic guidewire was carefully advanced from the left atrium, across the degenerated mitral valve into the left ventricle, and across the aortic valve into the descending aorta. A 6-F multipurpose catheter was introduced via the femoral artery. An Amplatz Goose Neck snare (Medtronic) was advanced through the multipurpose catheter and used to capture the distal end of the guidewire in the descending aorta. The wire was snared and externalized through the right femoral artery, creating a continuous rail: right femoral vein → right atrium → left atrium → mitral valve → left ventricle → aorta → right femoral artery. The interatrial septum was predilated using a 16-mm balloon to facilitate valve passage (Figure 3C, Video 3). However, the delivery system prolapsed into the huge left atrium, failing to engage the bioprosthetic valve. With reinforced tension applied to both ends of the rail, the system was straightened and advanced through the bioprosthetic valve (Figure 3D, Video 4). A 23-mm SAPIEN 3 valve (Edwards Lifesciences) (with +1 mL volume for optimal sealing) was deployed under rapid pacing (Figures 3E and 3F). Postdeployment TEE showed only mild paravalvular leak and no left ventricular outflow tract obstruction (Figure 4, Video 5).

Immediate hemodynamic improvement was noted: left ventricular end-diastolic pressure decreased to 12 mm Hg and mean left atrial pressure dropped to 15 mm Hg. The patient was extubated in the hybrid suite. Postprocedural TTE at discharge showed a mean transmitral gradient of 4 mm Hg and a mean left ventricular outflow tract gradient of 9 mm Hg. She was discharged on postprocedure day 3.

## Follow-Up

At 6-month follow-up, she was in NYHA functional class I, with TTE confirming stable valve function and significantly improved cardiac output.

## Discussion

This case underscores a critical technical challenge in mitral ViV implantation: failure of coaxial delivery due to massive atrial enlargement. In such patients, the giant atrium acts as a “wind sock,” absorbing the forward force from the delivery catheter and preventing engagement with the degenerated bioprosthetic valve. A slightly high transseptal puncture height and the extremely large septal-annular angle (106°) further compound this difficulty.

During the transcatheter mitral ViV procedure, transseptal puncture was technically crucial and challenging. For our case, the interatrial septum had been surgically incised and sutured during initial surgery, resulting in septal thickening. In addition, because of marked biatrial enlargement, a lower puncture site could not be achieved. A higher puncture height made the advancement of the delivery system extremely difficult because of the smaller angle between the inferior vena cava–atrial septum line and the mitral valve–left ventricular apex axis. In clinical practice, multiple transseptal puncture attempts are frequently performed to obtain a lower puncture height, and several puncture techniques may be used during this process. For example, a manually shaped puncture needle with dual curvature (distal and mid-shaft) may facilitate inferior puncture in a large atrium. Furthermore, the use of a larger outer sheath to support the puncture system may be helpful.

To address difficulty in advancing the delivery system during transcatheter mitral ViV implantation, several maneuvers can be attempted: predilating the septum with a balloon (even with an 18-mm balloon), balloon “massage” (gentle push-pull during inflation), preshaping of the stiff wire according to the atrial curvature, or creating a full-body arteriovenous rail. In refractory cases, conversion to an alternative access route (eg, transapical) may be required.

The full-body rail (arteriovenous loop) technique, borrowed from complex paravalvular leak closure, ventricular septal defect interventions, and percutaneous closure of congenital fistula,^2^ provides a definitive solution. By creating a rail with adequate support from the femoral vein to the femoral artery, we achieved enough tension. Bilateral tension straightens the cardiac axis, aligning the delivery system with the mitral valve plane. The rail prevents the system from prolapsing back into the atrium, which allows the valve to follow a rigid, predictable path. Although this technique requires arterial access and snaring, it is safe and avoids the need for transapical puncture^3^ or surgical conversion in frail patients. Predilation of the septum with a 16-mm balloon was also crucial in reducing friction on the system.

## Conclusions

In patients with massive biatrial enlargement and adverse septal-annular angles, the full-body rail technique is a safe and effective bailout strategy for transfemoral transseptal mitral ViV implantation. Interventional cardiologists should be proficient in this maneuver to ensure procedural success in complex anatomies.

## Funding Support and Author Disclosures

This study was supported by grants from the Shanghai Committee of Science and Technology, China (24SF1904804, 24SF1902004, and 25SF1902404). The authors have reported that they have no relationships relevant to the contents of this paper to disclose.

## Appendix

Video 1 Transesophageal Echocardiography Showing Bioprosthetic Leaflet Tear and Prolapse With Severe Intraprosthetic Regurgitation

Video 2 Left Ventriculography Showing Massive Mitral Regurgitation

Video 3 Predilation Using a 16-mm Balloon With an Arteriovenous Loop

Video 4 Cine Fluoroscopy Showing the Advancement of the Valve After Establishing the Rail

Video 5 Mild Paravalvular Leak on Postdeployment Transesophageal Echocardiography
